# Design of a Semi-Continuous Selective Layer Based on Deposition of UiO-66 Nanoparticles for Nanofiltration

**DOI:** 10.3390/membranes8040129

**Published:** 2018-12-12

**Authors:** Goji Y. Shangkum, Patchanee Chammingkwan, Dai X. Trinh, Toshiaki Taniike

**Affiliations:** Graduate School of Advanced Science and Technology, Japan Advanced Institute of Science and Technology, 1-1 Asahidai, Nomi, Ishikawa 923-1292, Japan; s1620414@jaist.ac.jp (G.Y.S.); chamming@jaist.ac.jp (P.C.); trinhxuandai@gmail.com (D.X.T.)

**Keywords:** metal–organic framework, UiO-66, composite membrane, water filtration, nanoparticle

## Abstract

Deposition of UiO-66 metal–organic framework nanoparticles onto a porous polymer support is a promising approach to designing highly-permeable, size-selective, flexible, and stable membranes for water filtration. In this article, a series of UiO-66 nanoparticles having different particle sizes were synthesized and employed to prepare UiO-66-deposited composite membranes. It was found that the size of the UiO-66 nanoparticles had great influences on the performance of the composite membranes for the filtration of a methylene blue aqueous solution. The deposition of smaller nanoparticles afforded a selective layer having a greater external surface area and narrower interparticle voids. These features made the deposition of smaller nanoparticles more advantageous in terms of the flux and rejection, while the deposition of greater nanoparticles afforded a selective layer more tolerant for fouling. Bimodal composite membranes were prepared by depositing mixed UiO-66 nanoparticles of smaller and bigger sizes. These membranes successfully combined the advantages of nanoparticles of a distinct size.

## 1. Introduction

Clean water scarcity is one of the serious global challenges, which is a consequence of exponential population growth, drastic climate change, and rapid industrialization. Among water purification technologies, membrane-based filtration plays a vital role in accessing superior water quality with an integrated sustainability in terms of no chemical additives, low energy consumption, minimal land usage, and ease of operation [1]. Filtration is generally classified into microfiltration, ultrafiltration, nanofiltration, and reverse/forward osmosis [2] according to the size of the solute to be rejected, in which the use of polymeric membranes is predominant due to certain advantages. For example, the mechanical flexibility of polymeric membranes is suitable for large-scale roll-to-roll processing and integration in advanced filtration modules. A range of pore forming techniques enable the fabrication of desired pore networks at a relatively low cost compared to an inorganic equivalent, and so on [3,4]. On the negative side, polymeric membranes tend to suffer from insufficient resistance to fouling, mechanical instability in the process stream, and a tradeoff between the permeability and selectivity [5,6,7]. One of the approaches to tackle these problems is hybridization of polymeric membranes with other materials, especially nanomaterials [4,8,9,10].

Recently, nanochannel materials have attracted increasing attention in the field of membrane-based filtration. The attraction comes from the surface-dominated behavior of a fluid in nanochannels, which is different from that of the bulk fluid [11]. For instance, a high surface atomic density and a low corrugation explain a nearly friction-less flow in nanochannels of multiwall nanotubes and graphitic materials [11,12,13]. Stacked graphene oxide offers ideal capillaries for facile transport of monolayered water molecules as horizontally spanned interlayer spaces of ~7 Å [14], while a disorder of the stacking offers a vertical pathway for the permeation [15]. Fast transportation with molecular-level selectivity through well-defined channels makes nanochannel materials a promising candidate to address the permeability–selectivity tradeoff problem of the conventional membranes.

Among various nanochannel materials, metal–organic frameworks (MOFs) deserve special attention as they equip exceptionally high porosity, tunable pore size, and structural diversity [16,17,18]. Contrary to rigid crystallographic structures for other porous materials, MOFs possess a flexible framework that can be tuned by selection of organic ligands and metal ions/clusters, thus making them particularly attractive for separation and purification purposes [19]. Among the preceding attempts to integrate MOFs into flexible polymeric membranes for water purification [20,21,22,23,24], a major challenge can be identified as how to form an MOF-based selective layer on a heterogeneous support in a defectless fashion. Li et al. [23] fabricated a continuous thin layer of a Zeolitic Imidazolate Framework-8 (ZIF-8) on a polyethersulfone (PES) support by impregnating PES with an aqueous solution of the metal precursor and subsequently interacting it with the organic ligand. As the reagents reacted at the interface of the aqueous and organic phases, ZIF-8 nuclei were formed and grown within pores to form a thin selective layer on the membrane surface. Zhang et al. [24] employed a coordination-driven in situ self-assembly method to fabricate ZIF-8/poly (sodium 4-styrenesulfonate) (PPS) selective layer on a polyacrylonitrile (PAN) support. It started from the coordination of the Zn^2+^ metal precursor with carboxylate groups of the hydrolyzed PAN substrate and dipping the Zn^2+^-coordinated substrate in a mixed solution of the organic ligand and PPS to form a composite overlayer that consisted of in situ generated ZIF-8 nanoparticles uniformly dispersed in the PPS matrix. The resultant membrane showed an excellent permeability by possessing a flux value of 265 L/m^2^ h MPa with efficient rejection of methylene blue (MB). In spite of promising performance, a key issue is that the nucleation and growth of MOFs usually requires hours under thermal treatment and/or in the presence of a harsh solvent [25], and this severely limits the combination between an MOF and a polymeric support that can form a uniform selective layer before damaging the polymeric support. Moreover, as the nucleation of MOF crystals likely occurs in a solution rather than on a substrate surface, it is not easy to control the thickness of the selective layer in in situ processes unless a cyclic layer-by-layer technique is employed [24,26]. In a practical sense, a facile and scalable process that can control the formation of a selective layer without deteriorating the substrate is required to fully exploit the advantageous of MOFs for water purification.

In our recent publication, a novel strategy was proposed to form an MOF-based composite membrane for nanofiltration [27], in which a suspension of UiO-66 nanoparticles as one of the most stable frameworks in water [28,29,30] was deposited onto a regenerated cellulose (RC) support via suction filtration. The nanoparticles were filled in the pore network or loaded on the external surface of the support membrane to form a selective layer. This new type of composite membrane exhibited an excellent permeability of 785.8 L/m^2^ h and almost 100% rejection in the filtration of a MB aqueous solution, as a consequence of dominant permeation through the selective nanochannels of UiO-66 with respect to the non-selective leakage through interparticle voids. Even though the selective layer was comprised of discontinuous agglomeration of the nanoparticles, it was indeed regarded semi-continuous in the filtration.

On the basis of the developed strategy, we have herein investigated the following issues of the new type of the composite membrane:

(i) The effect of the size and loading of the UiO-66 nanoparticles on the performance of the resultant composite membranes for the filtration of an MB aqueous solution. It was found that smaller nanoparticles yielded a selective layer superior in terms of permeability and selectivity, while a selective layer made by larger nanoparticles was more tolerant for the fouling.
(ii) Design of bimodal composite membranes with the selective layer composed by a mixture of smaller and larger nanoparticles. By filling interparticle voids among the larger nanoparticles with the smaller nanoparticles, the permeability, selectivity, and tolerance for the fouling could be simultaneously improved.

## 2. Materials and Methods

### 2.1. Materials

Zirconium tetrachloride (ZrCl_4_, 99%) and terephthalic acid (99%) were purchased from Sigma–Aldrich Co. (St. Louis, MO, USA) and were used as received. *N*,*N*-dimethylformamide (DMF) and methanol (MeOH) as solvents were purchased from Wako Chemical Industries Ltd. (Osaka, Japan). A regenerated cellulose (RC) membrane (RC58, diameter 47 mm, pore size 0.2 µm, Whatmann G.E. Healthcare, Tokyo, Japan) was used as a support membrane. Methylthioninium chloride (methylene blue (MB), 98.5%) was purchased from Kanto Chemical Co., Inc. (Tokyo, Japan) and was used as a solute for filtration experiments. Deionized (DI) water was used throughout the experiments.

### 2.2. UiO-66 Preparation and Characterization

Under nitrogen atmosphere, a solution of ZrCl_4_ (1.63 mmol in 30 mL of DMF) was added to a solution of terephthalic acid (2.28 mmol in 30 mL of DMF). Thereafter, a specified amount of DI water (0.2–2.0 mL) was added at once. The mixture was heated at 100 °C for 12 h under constant stirring and nitrogen atmosphere to yield a white dispersion of UiO-66 nanoparticles. The nanoparticles were collected by centrifugation and repetitively washed with 50 mL of DMF for three times. Thereafter, the solid product was extensively washed with MeOH to completely remove residual DMF and finally dried at 90 °C for 24 h. Thus, obtained samples were named UiO1–UiO5, which correspond to the addition of DI water volume of 2.0, 1.6, 0.8, 0.4, and 0.2 mL, respectively.

The crystalline structure of UiO-66 nanoparticles was analyzed by X-ray diffraction (XRD, Rigaku SmartLab, Tokyo, Japan) using Cu Kα radiation in the range of 2*θ* = 5–35° at the step of 0.1° per 150 s. The crystallite size (D) of UiO-66 was estimated using Scherrer’s equation:

(1) $$D=\frac{0.94\lambda }{\beta \cos\theta }$$

where *λ* = 1.54 Å for Cu Kα, *β* = full-width at half-maximum, and *θ* = Bragg’s angle. Fourier transform infrared spectra (FTIR) were acquired on a Jasco FT/IR-6100 spectrometer (Tokyo, Japan) in the range of 400–2400 cm^−1^ with a resolution of 4 cm^−1^. A dried sample was diluted with potassium bromide powder, and then pressed into a pellet for the measurement in the transmission mode. The morphology of UiO-66 nanoparticles was observed on a transmission electron microscope (TEM, Hitachi H7100, Tokyo, Japan) at an accelerated voltage of 100 kV. A dried sample was dispersed in MeOH, deposited onto a copper grid by drop casting, and then dried under air to evaporate the solvent. The particle size was acquired based on the TEM image analysis using ImageJ software. The thermogravimetric analysis (TGA) was performed on a Ragaku ThermoPlus EVO II thermal analyzer (Tokyo, Japan). A sample was loaded in an aluminum pan, then heated to 200 °C and kept for 2 h to remove water and residual MeOH. Thereafter, the temperature was raised to 600 °C at a heating rate of 5 °C/min under dry air. The organic content was reported based on the weight loss in the range of 300–600 °C relative to ZrO_2_ as a final residual.

### 2.3. Membrane Preparation and Characterization

A composite membrane was prepared by suction filtration of a dispersion of UiO-66 nanoparticles in water. An RC membrane was placed on a filter glass holder and wet with DI water. Then, 4.0 mg of UiO­66 nanoparticles (unless stated) was dispersed in 30 mL of DI water by sonication for 1 h, and then deposited on the RC membrane by suction filtration at a constant differential pressure of 50 mbar. After 30 min of filtration, the differential pressure was increased to 100 mbar and kept for 15 min to partially dry the membrane.

The morphology of the prepared composite membranes was observed by a scanning electron microscope (SEM, Hitachi S­4100, Tokyo, Japan) operated at an accelerated voltage of 20 kV. A sample piece was attached on a carbon tape and subjected to Pd/Pt sputtering for 100 s prior to the measurement. The cross-sectional morphology was also observed, where a membrane sample was fractured using liquid nitrogen.

### 2.4. Filtration Performance

The filtration performance of the composite membranes was evaluated based on the MB rejection from its aqueous solution (1.0 µM) at the constant differential pressure of 100 mbar. The concentrations in the feed (*C*_0_) and permeate (*C_p_*) solution were measured by UV/Vis spectroscopy (Jasco V670, Tokyo, Japan) based on the absorption intensity at 665 nm. The permeate solution was collected at the interval time of 1 or 2 min and the rejection (*R*) was calculated using Equation (2):

(2) $$R\;\left(\%\right)=\left(\frac{C_{0}-C_{p}}{C_{0}}\right)\times 100$$

The flux was determined from the cumulative permeate volume at the specified filtration time as shown in Equation (3):

(3) $$J=\left(\frac{V}{A\times t}\right)$$

where *J* represents the flux (L/m^2^ h), *V* is the cumulative permeate volume, *A* is the effective membrane area (calculated as 9.61 cm^2^), and *t* is the filtration time. The results were obtained as an average from at least two filtration experiments using independently prepared membranes.

## 3. Results

### 3.1. UiO-66 Characterization

**Figure 1:**
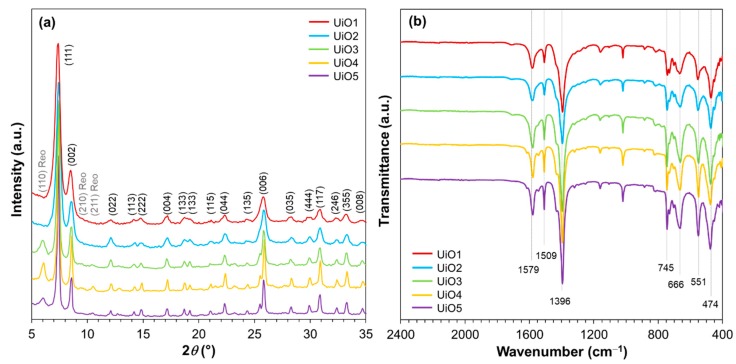
(**a**) XRD patterns; (**b**) FTIR spectra of UiO-66 nanoparticles.

A series of UiO-66 samples were prepared by varying the addition amount of water during the synthesis at the fixed molar ratio between ZrCl_4_ and terephthalic acid. X-ray diffraction patterns were recorded to confirm the formation of UiO-66. As illustrated in Figure 1a, all of the samples exhibited the intense diffraction peaks at 2*θ* = 7.4° and 8.5°, corresponding to (111) and (002) planes of the UiO-66 crystal [31,32,33,34]. Other minor diffraction peaks (as assigned in Figure 1a) also agreed well with the face-centered cubic lattice of the UiO-66 crystal [34]. By increasing the water amount, the diffraction peaks became broader, which is an indication of the crystallite size reduction. The crystallite sizes that were derived from Scherrer’s equation (Table 1) monotonously increased from 25 to 83 nm by decreasing the water amount. Minor and broad diffractions at 2*θ* = 5.9°, 9.6°, and 10.6° were additionally observed for UiO3, UiO4, and UiO5. These diffractions were attributed to the secondary crystalline phase, called the reo-type, which originated from defective nanodomains with missing clusters [35]. The functional groups present in the prepared UiO-66 samples were examined by FTIR. As illustrated in Figure 1b, all of the samples exhibited vibrational characteristics typical for UiO-66. Two strong bands at 1396 and 1579 cm^−1^ were assigned to the symmetric and asymmetric stretchings of O–C–O in the carboxylate groups of the terephthalic acid ligand, respectively [33,36]. The band at 1509 cm^−1^ represents the C=C vibration from the aromatic ring [37]. The characteristic peak at 745 cm^−1^ is ascribed to a mixture of the OH and C–H vibrational bendings [33] and the bands at 666, 551, and 474 cm^−1^ are assigned to the μ_3_-O stretching, Zr(O–C) stretching ,and μ_3_-OH stretching, respectively [33].

**Figure 2:**
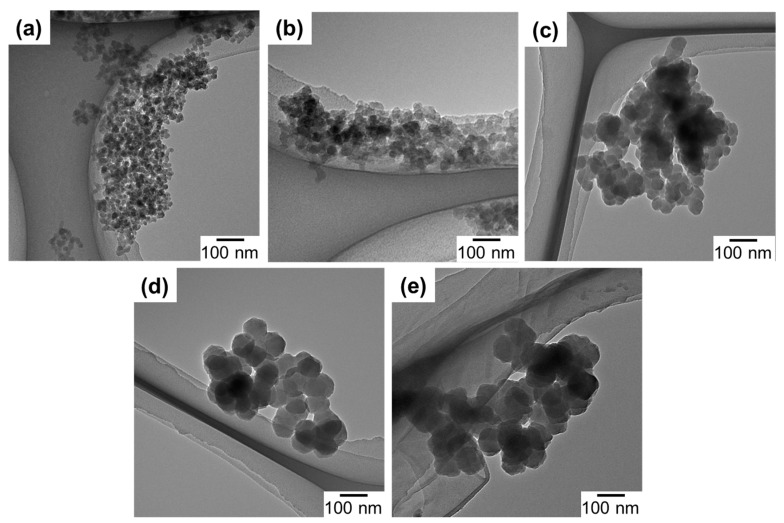
TEM images of UiO-66 nanoparticles: (**a**) UiO1; (**b**) UiO2; (**c**) UiO3; (**d**) UiO4; (**e**) UiO5.

The morphology of UiO-66 nanoparticles was observed by TEM and the particle sizes acquired from the TEM images are listed in Table 1. As can be seen in Figure 2, all of the samples exhibited a similar morphology, i.e., intergrown particles of a polygonal shape. The particle size monotonously increased by the decrease of the water amount, in accordance with the XRD results. A slight deviation between the crystallite and particle sizes plausibly originated from the polygonal shape and/or multiple crystal domains of nanoparticles. The dependence of the particle size on the water amount indicated the role of water to accelerate the formation of crystal nuclei. Ragon et al. [38] reported that the crystallization of UiO-66 became significantly faster in the presence of water, which was ascribed to the ease of the Zr_6_-cluster formation. Indeed, the reaction mixture became turbid more rapidly when a larger amount of water was added. The organic content of UiO1-5 (based on the weight loss in the TGA, Figure S1) is listed in Table 1. The theoretical weight loss between 300–600 °C for a perfect framework of 12 ligands per Zr_6_O_4_(OH)_4_ cluster was calculated as 54.6 wt% relative to ZrO_2_ as a final residual [33]. From the TGA result, the weight loss of UiO1 slightly deviated from the theoretical content, corresponding to 11 ligands per inorganic cluster. This slightly lower content was attributed to a missing linker defect that occurred randomly in the framework. On the other hand, the deviation became larger for a lower water addition amount. Especially for UiO4 and UiO5, the lower organic content also coincided with the formation of the reo topology observed from XRD, where each cluster was connected by eight linkers [35]. It was plausible that a smaller amount of water promoted the formation of the missing linker/cluster defect as a result of slower kinetics.

### 3.2. Membrane Preparation and Filtration Performance

**Figure 3:**
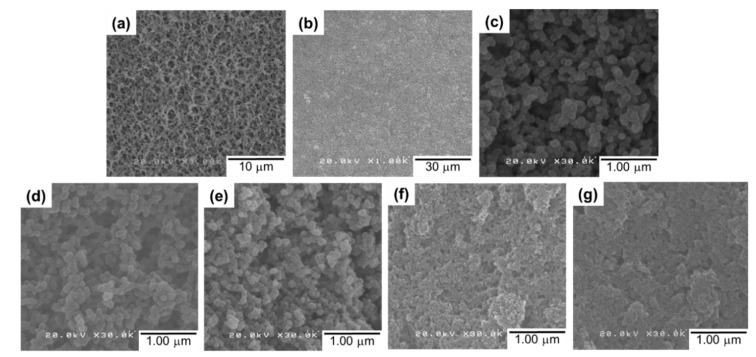
Top-view SEM images of the composite membranes: (**a**) a regenerated cellulose substrate; (**b**,**c**) UiO5 at different magnifications; (**d**) UiO4; (**e**) UiO3; (**f**) UiO2; (**g**) UiO1.

A series of UiO-66-deposited composite membranes were prepared by depositing 4.0 mg of preformed UiO1–UiO5 nanoparticles. Water was selected as a dispersing medium, which never damages the morphology of the support membrane. A top-view image of the RC membrane exhibited a highly-porous morphology (Figure 3a), which became invisible after the nanoparticle deposition (Figure 3b). Scanning electron microscope images of the UiO5 membrane (Figure 3b,c) revealed that the thin layer was made of the nanoparticles which were disorderly deposited without the formation of cracks and pinholes. A similar morphology was observed for the deposition of the other smaller nanoparticles, except the fact that the surface corrugation became more microscopic as the particle size became smaller (Figure 3d–g).

**Figure 4:**
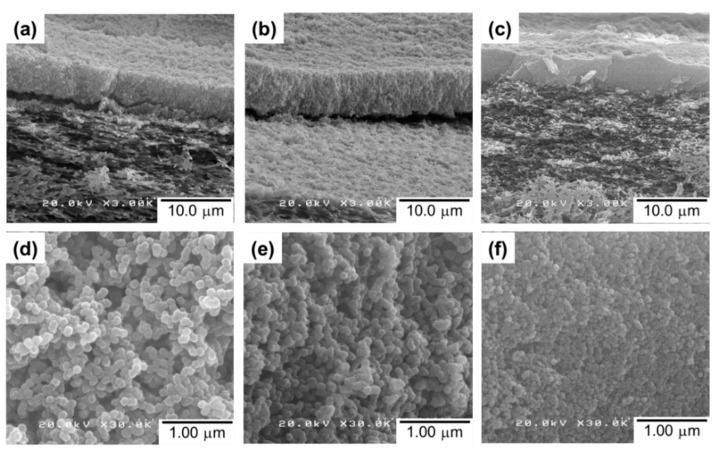
Cross-sectional SEM images of the composite membranes: (**a**,**d**) UiO5; (**b**,**e**) UiO4; (**c**,**f**) UiO2 at different magnifications.

The cross-sectional SEM images of the UiO5, UiO4, and UiO2 membranes (Figure 4a–c, respectively) showed the formation of a thin layer of nanoparticles (approx. 6 µm) on top of the porous support. The intrusion of the nanoparticles into the porous network of the support membrane was hardly observed, and it was similar for the other two samples. This was plausibly because of the intergrown morphology of the nanoparticles: the interconnected nanoparticles were not separable as isolated particles, and therefore, plugged the porosity of the membrane during the deposition so as to form a semi-continuous deposition layer. Magnification of the cross-section of the thin layer (Figure 4d–f) illustrated that the disordered packing of the nanoparticles generated interparticle voids, whose dimension was enlarged along the particle size. The thickness of the deposition layer was hardly affected by the particle size, suggesting that the packing density, i.e., the void fraction, was similar among the samples.

**Figure 5:**
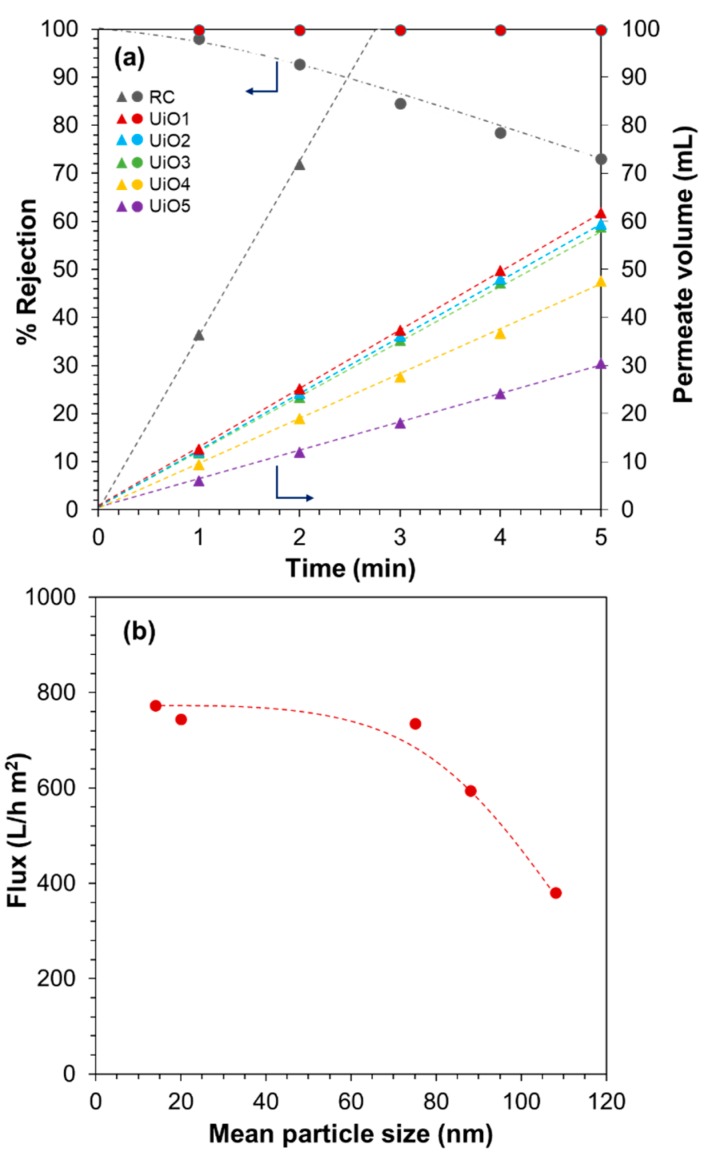
Filtration performance of UiO-66 composite membranes: (**a**) methylene blue rejection and permeate volume; (**b**) flux as a function of the UiO-66 particle size.

The filtration performance of the composite membranes was evaluated based on the rejection of MB from its aqueous solution. Figure 5a shows the results of the filtration in 5 min, where the RC membrane was used as a reference. The RC membrane exhibited the fastest permeation, but the rejection quickly dropped to 70% within 5 min owing to its pore size (0.2 µm) far larger than the molecular size of MB [39]. Relatively high rejection at 1 min (i.e., the first 50 mL of the permeate volume) was explained by the adsorption of MB to cellulose [27]. After consuming the adsorption capacity, the rejection quickly dropped. The deposition of UiO-66 nanoparticles greatly improved the filtration performance: the perfect rejection was maintained in 5 min for all of the samples, irrespective of the nanoparticle size. This fact supported the absence of the pinholes in the deposition layer as well as the effectiveness of the idea to form a selective layer by packing UiO-66 nanoparticles. The permeate volume increased linearly by time, indicating that the fouling by rejected MB molecules did not happen in 5 min. The permeation flux was found to be dependent on the size of UiO-66 nanoparticles (Figure 5b). The flux tended to increase for smaller nanoparticles, even though the dimension of interparticle voids became smaller. From N_2_ adsorption/desorption isotherms at 77 K (Figure S2), UiO1 and UiO5 nanoparticles were found to be predominantly microporous. Their mode pore sizes of 0.63–0.65 nm were similar among each other and consistent with the literature for tetrahedral pore structure of UiO-66 [40]. The interconnected morphology led to the formation of interparticle voids, whose dimension was comparable to the corresponding primary particle size: UiO1 formed the interparticle voids in the mesopore dimension (confirmed by the presence of a hysteresis loop), the voids for UiO5 were in the range of macropores (no hysteresis loop), and so on. These results also suggested that the microscopic corrugation of the surfaces of the deposition layer (rather than the pore features) led to an enlargement of the effective membrane area, as is the case for cross-linked polyamide composite membranes having the crumpled textures [41,42]. The saturation of the flux below 75 nm (i.e., UiO3 to UiO1) might be explained by the presence of a counter factor such as concave hydrophobicity interfaces [43], the tortuosity of the flow path, etc. The flux for UiO1 was calculated as 7734 ± 44 L/m^2^ h bar, which was close to our previous value [27], and it was 20–500 times greater than those of commercial membranes for ultra/nanofiltration such as polyvinylidene fluoride (100–300 L/m^2^ h bar) [44] and UTC-20 (15 L/m^2^ h bar) [45]. The flux values reported were 246 L/m^2^ h bar for a graphene oxide composite membrane [46] and 80–100 L/m^2^ h bar for cross-linked graphene oxide membranes [47]. There have been many studies which have reported on the utilization of MOFs for water purification, in most of which MOF nanoparticles were embedded in a polymeric selective layer as a permeability enhancing filler. A few studies employed MOFs as the main constituent of the selective layer. In one study [48], a polycrystalline UiO-66 selective layer was produced based on in situ seeding followed by secondary growth on an Al_2_O_3_ hollow fiber. The resultant membrane showed the permeability of 0.2–2.2 L/m^2^ h bar. In another two studies [49,50], UiO-66 nanoparticles or Zn–TCP nanosheets were deposited onto a support membrane by suction filtration from their dispersion. The resultant membranes showed the permeability of 2000–4000 L/m^2^ h bar, similar to ours in the order. The origin of the significantly different permeability values among the first study and the others (including ours) is at present unclear, while the external surface area may partly explain the difference. In the first study [48], the size of the in situ grown building blocks was around 2 μm, while in the other two [49,50] and our cases, nano-sized building blocks were used. Remembering that the deposition of 4.0 mg of UiO5 (size = 108 nm) led to the permeability of 380 L/m^2^ h and the equivalent gram of UiO1 (size = 14 nm) led to 770 L/m^2^ h, the external surface area obviously plays an important role for the permeability of the membranes.

**Figure 6:**
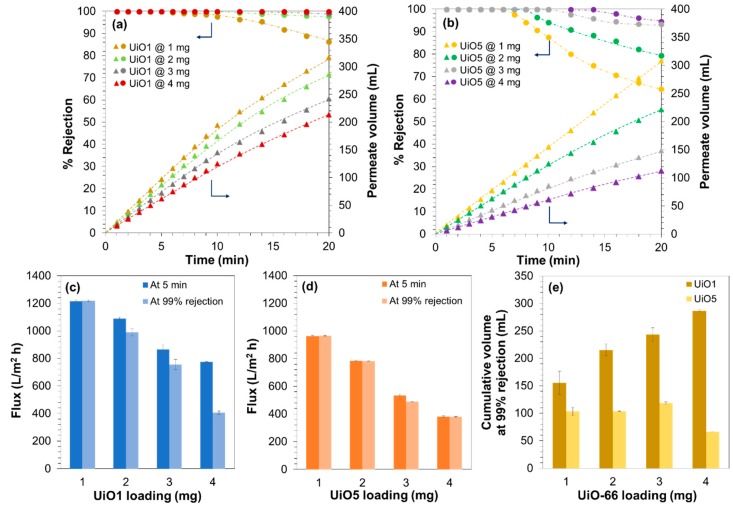
Filtration performance of the composite membranes with different amounts of UiO1 and UiO5: (**a**,**b**) time dependence of the filtration results for the first 20 min; (**c**,**d**) the permeability compared by the flux values at the first 5 min and at the timing of the 99% rejection; (**e**) the tolerance for the MB leakage estimated by the cumulative volume of the filtrate until which the rejection was kept over 99%.

In our previous work, it was revealed that the selectivity of UiO-66-deposited composite membranes originate from the molecular sieving ability of the intraparticle channels of UiO-66 [27]. Briefly, it was found that the adsorption capacity of the membrane was too small to explain the observed rejection, the deposition of poreless TiO_2_ nanoparticles instead of UiO-66 never improved the rejection, the observed cutoff between 1.22 and 2.28 nm was largely inconsistent with the dimension of the interparticle voids, and so on. Hence, deposited UiO-66 nanoparticles form a sort of semi-continuous selective layer, in which the passage of the filtrate occurs mainly through the intraparticle channels and that non-selective interparticle voids are negligible. In actuality, the leakage of the MB molecules through the interparticle voids and the membrane fouling were hardly observed at the filtration volume and time of Figure 5. On the other hand, it was interesting to investigate the tolerance of the composite membranes for the leakage and fouling in extended filtration experiments. For this purpose, the filtration experiment was continued until 350 mL of the feed solution was completely filtered. In the dead-end filtration setup, the progress of the separation monotonously increases the MB concentration in the remaining feed, so that this experiment aimed to identify potential defects of the composite membranes by exposing them to an increasing concentration of MB. The continuity of the performance at a constant MB concentration was also confirmed in separate experiments (Figure S3). Figure 6a,b describes the filtration results for a series of the composite membranes that were prepared by depositing 1–4 mg of UiO1 or UiO5 (see Figure S4 for the thickness variation along the loading). For up to 5 min, the permeate volume increased linearly to the time irrespective of the amount and size of the deposited UiO-66 nanoparticles. The flux values at 5 min are compared in Figure 6c,d. When the deposition amount was decreased from 4 mg, the flux value increased monotonously for both of UiO1 and UiO5, while UiO1 always retained the permeability greater than that of UiO5 at the individual loadings. Extended filtration over 5 min tended to deteriorate the permeability of the composite membranes due to the membrane fouling (Figure 6a,b). The extent of the deterioration was quite sensitive to the formulation of the membranes (Figure 6c,d). The flux values at the 99% rejection were almost unchanged from those of the first 5 min for UiO5, whilst the flux at the 99% rejection was at maximum halved from that of the first 5 min for UiO1. It was considered that microscopic concavity created by the deposition of UiO1 nanoparticles was more susceptive to the particle pore blocking from the accumulating solute. The extension of the filtration also caused the MB leakage through interparticle voids (Figure 6a,b). The leakage behavior was found to be opposite between the two kinds of the nanoparticles. The leakage tolerance improved monotonously to the deposition amount of UiO1, while the amount hardly suppressed the leakage for UiO5 (Figure 6e). The superiority of the UiO1-based membranes in terms of the permeability and selectivity appeared to be contradictive with a general tradeoff between these two parameters, but the following considerations would explain the obtained results: (i) smaller nanoparticles created a deposition layer with more microscopic corrugation, and thus, enhanced external surface area (contact area between the filtrate and the membrane) improved the permeability; (ii) the deposition of the smaller nanoparticles narrowed the interparticle voids, i.e., the pathway along which the leakage occurs. Besides, the larger interfacial area of the deposition layer (cf. Figure 4) suppressed a chance for the permeate to pass through the deposition layer without entering the nanochannels of UiO-66.

**Figure 7:**
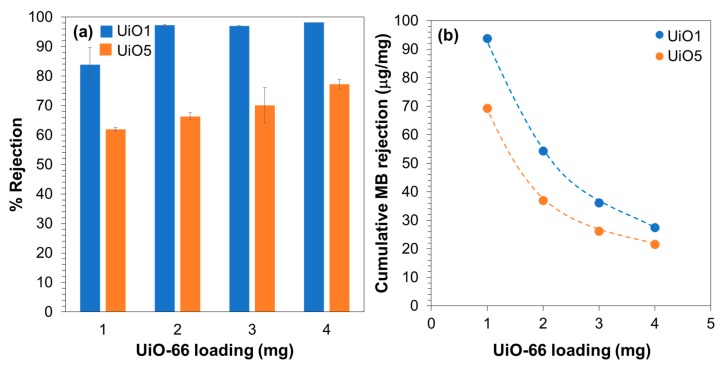
(**a**) Cumulative rejection for the filtration of 350 mL of the MB aqueous solution; (**b**) cumulative amount of MB rejected per loading.

In order to assure the origin of the MB rejection, an additional consideration was made based on the filtration results of 350 mL of the MB aqueous solution. The total amount of MB that was rejected by the filtration of the complete 350 mL was derived from the cumulative rejection (Figure 7a), and then, this amount was normalized by the loading of UiO-66 nanoparticles (Figure 7b). In our previous report, the MB adsorption capacity of UiO-66 nanoparticles having the particle size of 10 nm was determined as 2.8 µg/mg, where the nanoparticles were soaked in a MB solution for 42 h at room temperature and the adsorption capacity was derived from the reduced concentration of MB by the equilibrium adsorption [27]. The UiO-66 nanoparticles employed in this study were larger in size, so that 2.8 µg/mg could be regarded as the upper limit of the adsorption capacity of the UiO-66 nanoparticles. In Figure 7b, it is clear that the total rejection amount of MB was 10–30 times greater than the upper limit of the adsorption capacity, i.e., the MB rejection by the composite membranes was predominantly based on the molecular sieving mechanism [27]. A lower rejection efficiency at a greater loading suggested that not all of the UiO-66 nanoparticles were involved in the rejection, but an elevated loading was advantageous to fill in the interparticle voids.

**Figure 8:**
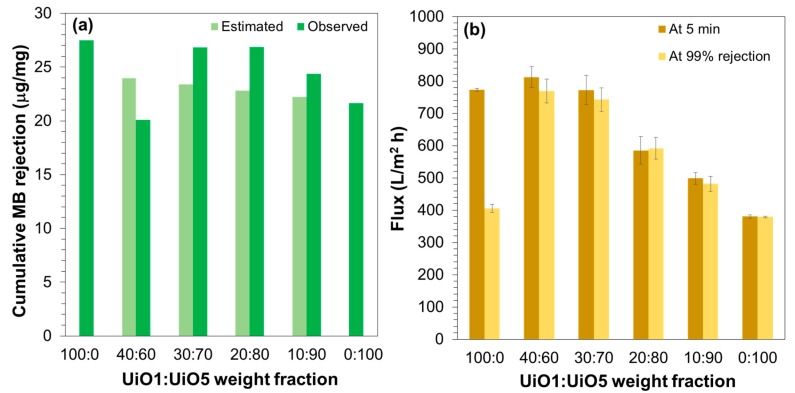
Filtration performance of bimodal composite membranes: (**a**) Cumulative rejection for the filtration of 350 mL of the MB aqueous solution; (**b**) permeability compared by the flux values at the first 5 min and at the timing of the 99% rejection. The bimodal composite membranes were prepared by depositing 4.0 mg of a mixture of UiO1 and UiO5 nanoparticles at different weight ratios.

As described above, the size of the UiO-66 nanoparticles exerted great impacts on the performance of the composite membranes: the smaller UiO1 nanoparticles yielded better permeability and tolerance for the MB leakage, while the larger UiO5 nanoparticles were resistant to the membrane fouling. At the end, we attempted to combine these advantages of the smaller and larger nanoparticles by depositing 4.0 mg of a mixture of UiO1 and UiO5 nanoparticles onto the support membrane (termed bimodal membranes). In a microscopic view, it was expected that a small weight fraction (but a much larger number) of the UiO1 nanoparticles would attach onto the surfaces of the UiO5 nanoparticles, and this would result in an enlarged external surface area of the selective layer and filled (or narrowed) interparticle voids. Figure 8a illustrates the cumulative MB rejection per loading for the complete filtration of 350 mL of the MB aqueous solution. It was found that the inclusion of a small weight fraction of the UiO1 nanoparticles notably improved the capacity of the membrane to reject MB. The extents of the improvement were much greater than those estimated from the weight average (except 40:60). Most plausibly, the interparticle voids among the UiO5 nanoparticles were filled by the UiO1 nanoparticles, and as a result, the interparticle voids became as narrow as those of the UiO1-based membrane (corresponding to 100:0). The exceptional result for 40:60 would be explained by the fact that the packing density of bimodally distributed particles reached its peak at 70% of the large particle fraction irrespective of the particle size ratio [51]. Figure 8b describes the permeability of the bimodal membranes measured at the first 5 min and at the timing of 99% rejection. As was expected, the inclusion of a small fraction of the UiO1 nanoparticles monotonously enhanced the permeability of the membranes, and the flux value became almost comparable at the weight ratio of 30:70. Furthermore, the usage of the UiO5 nanoparticles as the main component of the selective layer successfully endowed the fouling resistance of the UiO5-based membrane (corresponding to 0:100) to the bimodal membranes in a way not to sacrifice the permeability and the selectivity of the UiO1-based membrane.

## 4. Conclusions

The deposition of preformed nanoparticles onto a porous polymer support is a facile strategy to access a performant and flexible composite membrane having a semi-continuous selective layer of a metal–organic framework. In this article, a series of composite membranes were prepared by depositing UiO-66 nanoparticles onto a regenerated cellulose support, and the impacts of the size and its distribution of the nanoparticles were examined on the membrane performance for nanofiltration. Excellent permeability of 400–1200 L/m^2^ h and rejection of 22–94 mg of methylene blue per gram of UiO-66 were obtained, where the origin of the selective permeation was attributed to the nanochannels of UiO-66. At a fixed loading of UiO-66 nanoparticles, smaller nanoparticles tended to form a selective layer having better permeability and selectivity. The former was explained by a larger external surface area of the microscopically corrugated selective layer, and the latter was mostly due to the creation of narrower interparticle voids. The utilization of larger nanoparticles was inferior to smaller ones in terms of the flux and rejection, but it greatly suppressed the membrane fouling. When the UiO-66 nanoparticles having two different sizes were co-deposited at an appropriate weight ratio, the resultant selective layer was found to equip the best level of the permeability, selectivity, and fouling resistance. In conclusion, the present article successfully demonstrates promising aspects of the new type of MOF-based composite membranes for nanofiltration: excellent performance is realized on the basis of facile production and easy optimization through the size distribution of MOF nanoparticles that can be ex situ prepared.

## Figures and Tables

**Table 1:** Characteristics of UiO-66 nanoparticles.

Sample	Water Amount ^1^ (Molar Ratio)	Crystallite Size from XRD ^2^ (nm)	Particle Size from TEM ^3^ (nm)	Organic Content ^4^ (wt%)	Ligand per Zr_6_O_4_(OH)_4_ Cluster
UiO1	68 (2.0 mL)	25 (18)	14	54.09	11
UiO2	55 (1.6 mL)	37 (21)	20	51.84	10
UiO3	27 (0.8 mL)	46 (37)	75	51.08	10
UiO4	14 (0.4 mL)	66 (51)	88	46.28	8
UiO5	6.8 (0.2 mL)	83 (52)	108	46.83	9

^1^ Molar ratio of added water with respect to ZrCl_4_. The corresponding volume is shown in the parenthesis; ^2^ Determined using Scherrer’s equation based on the (002) plane. The values in the parenthesis were determined based on the (111) plane; ^3^ The median particle size acquired from TEM images using ImageJ software; ^4^ The reported values are the weight loss between 300–600 °C relative to ZrO_2_ as a final residual. Uncertainty in reproduction was within 0.2 wt%.

## Supplementary Materials

The following are available online at http://www.mdpi.com/2077-0375/8/4/129/s1, Figure S1: TGA profiles of UiO-66 nanoparticles, Figure S2: N_2_ adsorption/desorption results for UiO1 and UiO5 nanoparticles: (a) isotherms; (b) micropore size distribution, Figure S3: Performance of the UiO1 composite membrane in an elongated filtration, Figure S4: cross-sectional SEM images of the UiO5 composite membranes at different loadings: (a) 1.0 mg; (b) 3.0 mg; (c) 4.0 mg.
