# Supplementary material for: Design of a Semi-Continuous Selective Layer Based on Deposition of UiO-66 Nanoparticles for Nanofiltration
Source: Membranes (Basel). 2018 Dec 12;8(4):129. doi: 10.3390/membranes8040129 (PMC6315370; doi:10.3390/membranes8040129)
Supplement: Supplementary file 1 [file membranes-08-00129-s001.pdf]

membranes

Supplementary Information

Design of a Semi-Continuous Selective Layer Based on Deposition of UiO-66 Nanoparticles for Nanofiltration

Goji Y. Shangkum, Patchanee Chammingkwan, Dai X. Trinh and Toshiaki Taniike *

Graduate School of Advanced Science and Technology, Japan Advanced Institute of Science and Technology, 1-1 Asahidai, Nomi, Ishikawa 923-1292, Japan; s1620414@jaist.ac.jp (G.Y.S.); chamming@jaist.ac.jp (P.C.); trinhxuandai@gmail.com (D.X.T.)

* Correspondence: taniike@jaist.ac.jp; Tel.: +81-761-51-1630

Received: 20 October 2018; Accepted: 8 December 2018; Published: date

TGA profiles

The plateau in the range of 250-400 °C and the final residual at 600 °C corresponded to the chemical formula of $ZrO(CO_2)_2(C_6H_4)$ and ZrO_2, respectively. The organic content was reported based on the weight loss in the range of 300-600 °C relative to ZrO_2 as a final residual.

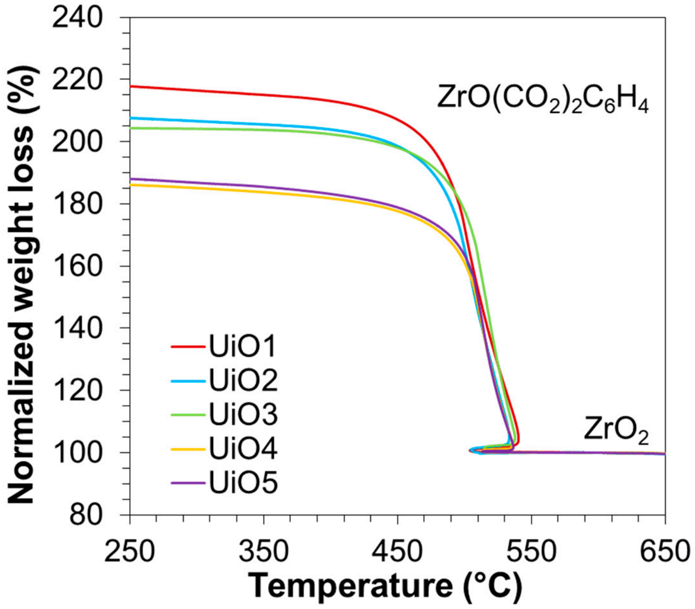

Figure S1. TGA profiles of UiO-66 nanoparticles.

N_2 adsortion/desorption measurement

N_2 adsorption and desorption isotherms of UiO1 and UiO5 were acquired at 77 K using a BELSORP-max instrument (BEL JAPAN, Inc.). 20-30 mg of a sample was charged into a sample cell and sealed with a brass filler and a rubber cap. The sample was outgassed at 150 ºC for 24 h *in vacuo* prior to the measurement. The BET surface areas of UiO1 and UiO5 were determined as 804 m^2/g and 1119 m^2/g, respectively. The micropore size distribution was calculated using the grand canonical Monte Carlo method using BELMaster7™ software.

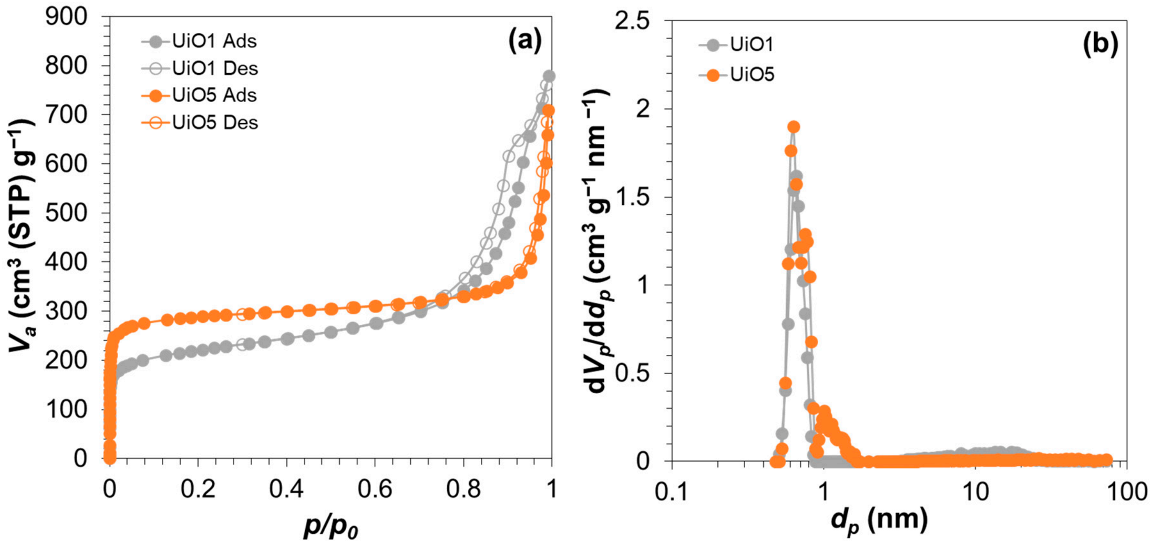

Figure S2. N_2 adsorption/desorption results for UiO1 and UiO5 nanoparticles: (**a**) Isotherms; (**b**) micropore size distribution.

Performance in elongated filtration

The performance of the composite membranes was investigated for a longer time of period, where an aqueous solution of MB of an appropriate concentration was continuously filled during the filtration in order to maintain the MB concentration of the feed solution at 1.0 µM. The result of the filtration is shown in Figure S3. It was found that nearly 100% rejection was maintained up to 2500 mL of the permeate and the duration over 400 min.

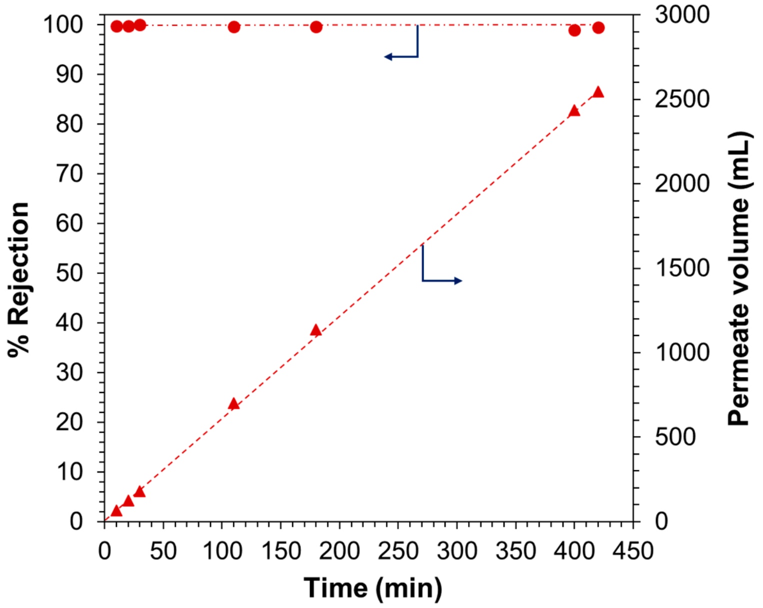

Figure S3. Performance of the UiO1 composite membrane in an elongated filtration.

Cross-sectional SEM images for UiO5 composite membranes at different loadings

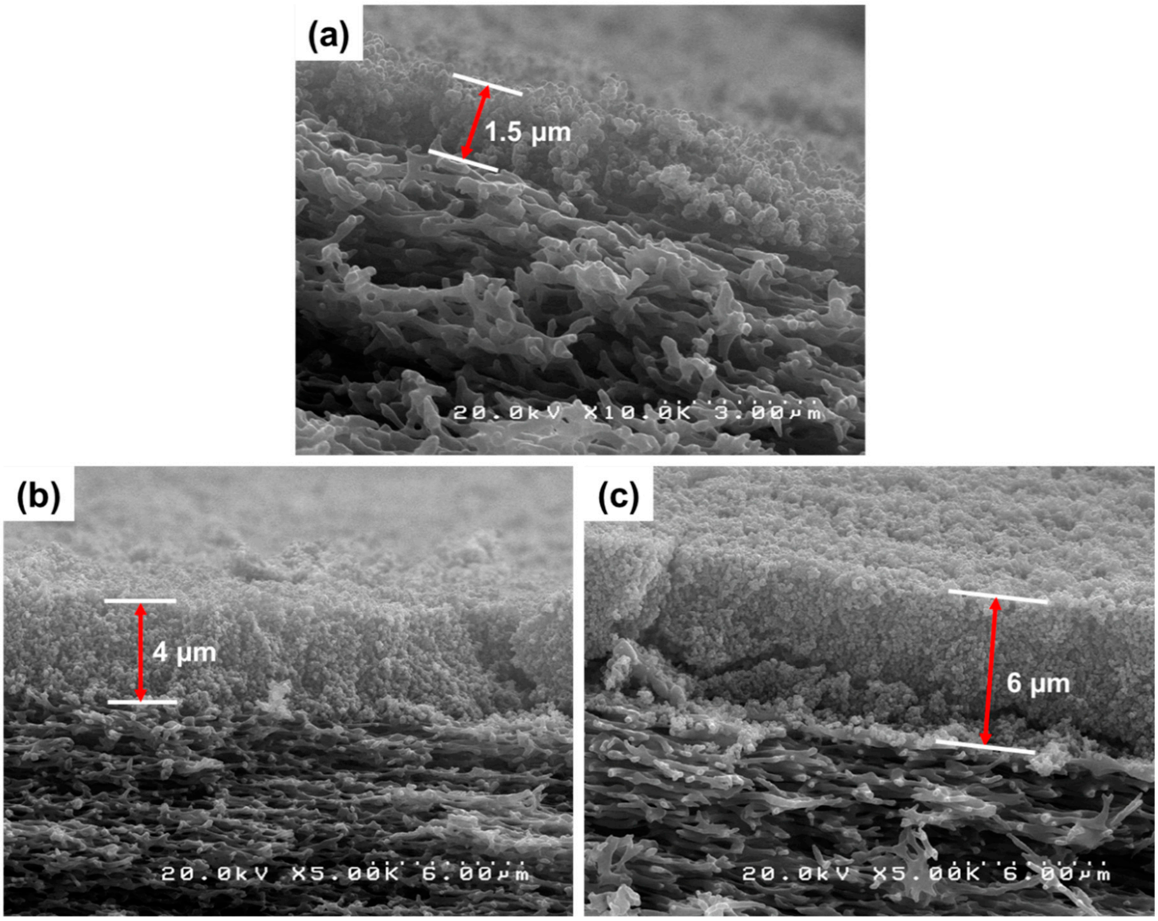

Figure S4. Cross-sectional SEM images of the UiO5 composite membranes at different loadings: (**a**) 1.0 mg; (**b**) 3.0 mg; (**c**) 4.0 mg.

© 2018 by the authors. Submitted for possible open access publication under the terms and conditions of the Creative Commons Attribution (CC BY) license (http://creativecommons.org/licenses/by/4.0/).
